# A randomized controlled trial of vitamin D supplementation on perinatal depression: in Iranian pregnant mothers

**DOI:** 10.1186/s12884-016-1024-7

**Published:** 2016-08-20

**Authors:** Farideh Vaziri, Samira Nasiri, Zohreh Tavana, Mohammad Hossein Dabbaghmanesh, Farkhondeh Sharif, Peyman Jafari

**Affiliations:** 1 Department of midwifery, School of Nursing and Midwifery, Shiraz University of Medical Sciences, Shiraz, Iran; 2 Student Research Committee, School of Nursing and Midwifery, Shiraz University of Medical Sciences, Shiraz, Iran; 3 Department of Obstetrics and Gynecology, Medical school, Shiraz University of Medical Sciences, Shiraz, Iran; 4 Shiraz Endocrinology and Metabolism Research Center, Shiraz University of Medical Sciences, Shiraz, Iran; 5 Community Based Psychiatric Research Center, Shiraz University of Medical Sciences, Shiraz, Iran; 6 Biostatics Department, School of medicine, Shiraz University of Medical Sciences, Shiraz, Iran

**Keywords:** Maternal depression, Vitamin D, Supplementation, Ante and postnatal depression, Pregnancy complication

## Abstract

**Background:**

Mood disorders in pregnancy and post-partum period are common and considered as a public health issue. Researchers have studied the relationship between low serum vitamin D concentration and perinatal depression, although no clinical trial has been conducted on vitamin D’s effects on depression related to childbirth. This study evaluated the effect of vitamin D_3_ supplementation on perinatal depression scores.

**Methods:**

This randomized clinical trial was done in pregnant women who were under prenatal care in a teaching hospital in Shiraz, Iran. The inclusion criteria were: being 18 years or older, no history of mental illness and internal diseases, a singleton live fetus, without any pregnancy complications, gestational age of 26–28 weeks upon enrollment, and depression score of 0 to 13. The Edinburgh Postnatal Depression scale was used to evaluate depression scores. A total of 169 participants were assigned to the two groups of placebo and vitamin D through block randomization design. Vitamin D group received 2000 IU vitamin D_3_ daily from 26 to 28 weeks of gestation until childbirth. Maternal serum 25-hydroxyvitamin D concentrations were measured at baseline and childbirth. Besides, depression scores were evaluated four times: at 26–28 and 38–40 weeks of gestation, and finally at 4 and 8 weeks after birth.

**Results:**

The two groups were similar in relation to baseline 25-hydroxyvitamin D concentrations. However, at childbirth, the vitamin D group had significantly higher 25-hydroxyvitamin D concentration in comparison to the control group (*p < 0.001*). At baseline, no correlation was observed between 25-hydroxyvitamin D concentration and depression score (*r* = 0.13, *p* = 0.09). There was no significant difference between the two study groups in relation to the baseline depression score. While, the vitamin D group had greater reduction in depression scores than the control group at 38–40 weeks of gestation (*p* = 0.01) also, at 4 and 8 weeks after birth (*p* < 0.001).

**Conclusions:**

The present trial showed that consuming 2000 IU vitamin D_3_ daily during late pregnancy was effective in decreasing perinatal depression levels. We suggest further clinical trial in pregnant mothers who are at risk for postnatal depression.

**Trial registration:**

Iranian Registry of Clinical Trials IRCT2015020310327N11. Date of registration: March 9th 2015.

**Electronic supplementary material:**

The online version of this article (doi:10.1186/s12884-016-1024-7) contains supplementary material, which is available to authorized users.

## Background

Mood disorders in pregnancy and post-partum period are common and considered as a public health issue. In recent decades researchers have paid great amount of attention to these subjects and showed that mood illness related to childbirth can lead to serious complications for mother, fetus, newborn and family [1, 2]. Evidence shows that postnatal depression is an extension of antenatal depression which continues postpartum. This problem can have negative effects on infant development and can cause behavioral, cognitive, social and emotional problems in infant which could continue throughout childhood [3–5].

Prevalence of postpartum depression varies throughout the world; however researchers have used different scales and cut off points to describe this psychological disorder. In a systematic review by Halbreich and Karkun, the studies from 40 different countries were reviewed. This study has reported the prevalence of postnatal depression up to 60 % [6]. The prevalence of postnatal depression in some Asian countries has been reported between 22 and 46.9 % [7, 8]. Several studies have shown a high prevalence of postnatal depression in Iran. In the meta-analysis of Veisani et al., postnatal depression was reported at 25.3 % [9], and in two recent studies in eastern and central part of Iran, the prevalence has been reported at 28.9–34.8 % [10, 11].

The spread and importance of postpartum depression have led researchers to create preventative methods such as educational programs, modified care, dietary supplements, antidepressant medication and social support [12–14].

In human body, the most important source of vitamin D is the skin where 7-dehydrocholesterol is converted to vitamin D_3_ in response to UV exposure. Both cutaneous production and dietary consumption of vitamin D must undergo two hydroxylations in the body for activation. Following the first hydroxylation in the liver, 25-hydroxyvitamin D is produced. However, 25-hydroxyvitamin D requires an additional hydroxylation in the kidneys to form the biologically active form of vitamin D, 1, 25(OH) 2D. Serum 25-hydroxyvitamin D as the main precursor of 1, 25(OH) 2D has a circulating half-life of 2–3 weeks. While, the circulating half-life of 1, 25(OH) 2D is around 4 h. Its blood concentration is 1000 times less than 25-hydroxyvitamin D. Serum levels of PTH, calcium, and phosphate have influence on regulatory and production of 1,25(OH)2D. Serum levels of 25-hydroxyvitamin D reflects the body’s vitamin D reserves and this is a well-known indicator for examining vitamin D status [15]. Several studies have revealed that vitamin D supplementation can raise vitamin D reserves gradually, according to baseline concentration of vitamin D, dose and duration of consumption [16–18]. In accordance to a guideline by Holick et al., 6–8 weeks consumption of a high dose vitamin D supplement are essential to obtain an adequate blood reserves of vitamin D [19].

It seems that in addition to calcium homeostasis and bone health, vitamin D is also essential for brain development and function [20, 21]. The special nuclear receptors for 1, 25-dihydroxyvitamin D and also essential enzymes for hydroxylation of vitamin D are found in the central nervous system. Therefore, the brain can regionally activate vitamin D, which implicates vitamin D’s role in brain function more prominent [22–25].

Studies have shown that low serum 25-hydroxyvitamin D concentration is associated with psychological symptoms like anxiety, depression and reduced cognitive function [26, 27]. Few case reports have suggested an interaction between season or latitude and psychological problems [28, 29]. In a study by Mozaffari- Khosravi et al., high doses of vitamin D have been effective in men and women who were depressed [30]. Li et al. suggested that more accurate clinical trials are required to reach a conclusion about the effectiveness of vitamin D on depression [31].

Considering the possible connection between low serum vitamin D and depression, researchers studied the relationship between low vitamin D and perinatal depression. Murphy et al. in a study with a small sample size (97 participants), observed an association between maternal low serum concentrations of 25-hydroxyvitamin D and high depression scores within several post-partum visits [32]. Robinson et al. studied the relationship between the maternal serum 25-hydroxyvitamin D concentrations measured in the 18^th^ week of gestation and postnatal depression in 796 people. They found that low serum 25-hydroxyvitamin D levels in the second trimester were a risk factor for postnatal depression. Depression scores were measured three days after childbirth using a questionnaire based on the Edinburgh Postnatal Depression scale (EPDS) [33]. Brandenburg et al. studied the association between maternal 25-hydroxyvitamin D concentration and antenatal depression. Serum 25-hydroxyvitamin D concentrations were measured in 4,236 pregnant mothers around their 13 weeks of gestation and their depression scores were measured at 16 weeks. It has been shown that mothers with higher depression scores had lower levels of 25-hydroxyvitamin D compared to who had lower depression scores [34]. On the contrary to the above mentioned studies, Neilson et al., in a case–control study, found that an increase in 25-hydroxyvitamin D level can enhance the possibility of postnatal depression. In this study, they selected mothers who needed antidepressant drugs after childbirth as the case group. The mothers who were not on antidepressant drugs were selected as the control group. They have stated that, these unexpected results could be due to genetic differences which lead to a disturbance in conversion of 25-hydroxyvitamin D into its active form, calcitriol [35].

According to our knowledge, there was no clinical trial that has evaluated the effectiveness of vitamin D on perinatal depression. Hence, we aimed this study to assess the effectiveness of vitamin D_3_ supplementation on antenatal and postnatal depression levels.

## Methods

### Setting and participants

This randomized clinical trial was done in pregnant women who were under prenatal care in Hafez hospital which is a tertiary hospital in Shiraz, Iran, affiliated to Shiraz University of Medical Sciences. Nulliparous and multiparous pregnant women were entered into this study. The inclusion criteria were: being 18 years or older, healthy women means; any one with no history of mental illness and internal diseases such as hyper/hypothyroidism, parathyroid, renal, diabetes and heart diseases, no addiction to any kind of narcotic drugs, living with a husband, a singleton live fetus, without any pregnancy complications such as preeclampsia, gestational diabetes, ruptured membranes and suspicion of preterm birth, no previous cesarean sections, gestational age of 26–28 weeks based on ultrasound results, and the EPDS baseline scores of 0 to 13. Exclusion Criteria were: the participants with the EPDS baseline scores of >13, not providing blood sample at the onset of the study, and less than 8 weeks consumption of vitamin D_3_ supplementations (for example due to preterm labor or stopping consumption), irregular consumption (for example every two/three days instead of daily consumption).

In this study, data collection lasted from November 2014 until early October 2015. The present study was approved by the Ethics Committee of Shiraz University of Medical Sciences with the ethics code 93–7212. Also, the present study has been recorded in the Iranian Registry of Clinical Trials’ website with the code number IRCT2015020310327N11.

### Data collection tools

In this study, we used the Edinburgh Postnatal Depression scale (EPDS) in order to evaluate depression levels. This questionnaire is comprised of ten multiple choice questions, the minimum score is 0 and the maximum is 30. Advantage of this questionnaire is that it focuses on psychological signs of depression rather than physical signs [36]. The EPDS was originally utilized in the postpartum period only. After that, several studies have validated its use throughout the antenatal period [37–39]. In western studies, this questionnaire has had high reliability and validity [40, 41]. It was translated to Persian and was used in the study by Mazhari and Nakhaee, with a validity and reliability of 0.76 and 0.83, respectively [42]. Cut-off points with values more than 9, 12, 13, and 14 were used in different studies to determine perinatal depression [37, 43, 44]. In the present study, we used the cut-off point of > 13 as greater likelihood of having depression disorder, according to Mazhari and Nakhaee’s study in Iranian population [42]. Therefore, at baseline we excluded those mothers with the EPDS scores of >13 and they were referred to a psychologist for further evaluation and management. The cut-off points of <9 and 9–13 were used as low and moderate risk for depression.

Serum 25-hydroxyvitamin D was measured with the Chemiluminescence immunoassay (CLIA) method [45]. After separating the serum from blood sample, the serum was kept under −20 °C temperature until the laboratory test was done. The vitamin D measurements were all done in one laboratory in Shiraz. The lab technicians were blind to group allocations.

According to the Institute of Medicine (IOM), we considered vitamin D deficiency as a serum 25-hydroxyvitamin D of less than 20 ng/ml. We also considered vitamin D insufficiency as a serum 25-hydroxyvitamin D of 21–29 ng/ml, and the participants who had serum 25-hydroxyvitamin D of ≥ 30 ng/ml as sufficient [19, 46].

### Design and data collection

At first, a research team member who was responsible for data collection visited the prenatal care clinic of the hospital daily and based on the inclusion criteria, invited the mothers to participate in the study. She clarified the study’s goals and if the mothers were willing to participation a written informed consent was obtained from them. The maternal baseline depression levels were evaluated. The mothers with depression score of 0–13 were entered in the study. Finally, the candidates were referred to the lab in order to provide blood samples for determining serum 25-hydroxyvitamin D concentrations.

Intervention in the supplement consumer (vitamin D) group: In 2007 and 2010, the Canadian Pediatric Association recommended a daily amount of 2000 IU of vitamin D for pregnant and breast-feeding women [47, 46]. Therefore, this group received two 1000 unit vitamin D_3_ pills (Totally 2000 IU) daily from the 26–28 weeks of gestation until childbirth. The pills were produced by the Jalinous pharmaceutical company in Iran. The consumptions of pills were assessed in later prenatal care visits and over the phone. Maternal blood samples were obtained at 26–28 weeks of gestation (at baseline) and once more, at childbirth (during first 24 h after birth in postpartum ward). Depression scores were determined four times: at 26–28 weeks of gestation (baseline), at 38–40 weeks of gestation, and finally at 4 and 8 weeks after birth. This group also received routine prenatal care.

Intervention in the control group: the control group received two placebo pills composed of starch daily from 26 to 28 weeks. Also, other study protocol measures for this group were similar to the vitamin D group. In Iran, iron and folic acid supplements are routinely given to all pregnant mothers and some mothers based on doctors’ order may consume multivitamins or calcium. In order not to deprive the participants from antenatal supplements all participating mothers were allowed to use prescribed supplementation outside this study’s protocol. The multivitamin supplementations contained 200–400 IU of vitamin D.

### Sample size and statistical analysis

We could not find a similar study to use its means for determining the sample size. Therefore, considering the following values: effect size = 0.5, α = 0.05, β = 0.20, to the power of 80 % and using the formula (16/∆^2^, ∆ = 0.05), the sample size was estimated at 64 in each group. Considering the possible losses, the sample size was set at 74 people in each group finally. Stratified random sampling was performed in this study. Nulliparous and multiparous mothers were considered as the strata. In the strata, each eligible pregnant woman was assigned in the control or the vitamin D groups using block randomization strategy. The data were analyzed using the SPSS statistical software (v. 16). Besides, *P*-values < 0.05 were considered as statistically significant. The normality of the data was evaluated using the Kolmogorov-Smirnov test. The means of normal distribution data were analyzed using parametric tests such as Student’s *t*-test or paired t- test. A non parametric test (Mann Whitney *U* test) was used whenever the continuous measures were not normally distributed. The distribution of serum 25-hydroxyvitamin D concentration at baseline and childbirth was not normal in the all participants. Therefore, a nonparametric test was used for 25-hydroxyvitamin D data.

We conducted stratified analyses by parity status to assess whether the trend of effectiveness is different among the two nulliparous and multiparous subgroups. And in order to determine changes over time, the repeated measures analysis was used. Correlations between continuous.

This was a single-blind study. Data collection related to depression, at 38–40 weeks of gestation, at 4 and 8 weeks after birth were done by a trained midwife outside the research team. She was blind to group allocations.

## Results

**Fig. 1:**
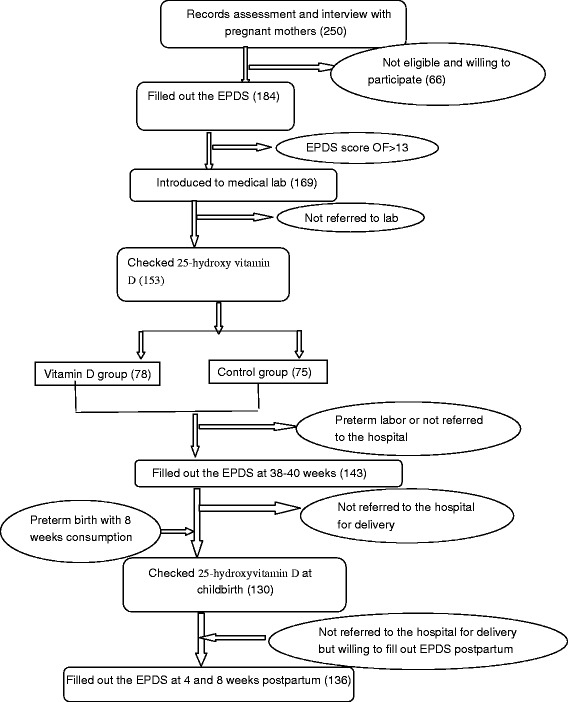
The flow chart of sampling

In this trial study, first 250 pregnant mothers were interviewed. Out of which, 184 qualified mothers were selected who completed the baseline depression questionnaire. Amongst them, 15 mothers leaved the study, because their depression scores were higher than 13. The Remaining 169 participants were assigned to the control or vitamin D groups with a block randomization design, and they were referred to the laboratory to provide blood sample. 16/169 women did not refer to the lab. At child birth, blood sample was collected for 130 mothers. Another significant factor for sample loss was that a number of the women did not return to the specified hospital for childbirth. Finally, 136 participants completed the EPDS at 4 and 8 weeks postpartum. The sampling flow chart was presented in Fig. 1.

The mean age of the participants was 26.31 ± 4.59. Age had a minimum and maximum value of 18 and 39, respectively. Of total participants 92 (60.1 %) mothers were nulliparous and 61 (39.9 %) were multiparous women. The two study groups were similar regarding to age, job, education, parity and sun exposure. However, they were different regarding to using other supplements outside the study’s protocol and planned pregnancy (Table 1).

**Table 1:** Comparison of demographic data in the intervention and control group

Variables	Vitamin D	Control	*p*-value
Age (year) Mean ± sd	26.40 ± 4.88	26.22 ± 4.33	0.80
Job N (%)			0.96
Housewife	72 (96)	75 (96.2)	
Employed	3 (4)	3 (3.8)	
Education N (%)			0.55
High school	26 (34.7)	21 (26.9)	
High school diploma	34 (45.3)	38 (48.7)	
University	15 (20.0)	19 (24.4)	
Parity			0.17
Nullipara	41 (54.7)	51 (65.4)	
Multipara	34 (45.3)	27 (34.6)	
Using others supplements			0.017
Yes	55 (73.3)	69 (88.5)	
No	20 (26.7)	9 (11.5)	
Planned pregnancy			0.01
Yes	55 (73.3)	69 (88.5)	
No	20 (26.7)	9 (11.5)	
Seasonal distribution in onset N (%)			0.32
Autumn	9 (12)	6 (7.7)	
Winter	11 (14.7)	7 (9)	
Spring	55 (73.3)	65 (83.3)	
Sun exposure(minutes)			0.55
< 15	14 (21.9)	12 (16.7)	
15–30	28(43.8)	33 (45.8)	
30–60	10 (15.6)	17 (23.6)	
> 60	12 (18.8)	10 (13.9)	

At baseline, 25-hydroxyvitamin D had a mean value of 12.35 ± 7.17 ng/ with a minimum and maximum value of 4 and 49.30 ng/mL, respectively. This variable had a median value of 11.20 and mode value of 4. Vitamin D deficiency (25-hydroxyvitamin D concentration of <20 ng/mL) was observed in 134 women (87.6 %). Furthermore, 15 women (9.8 %) had insufficiency (25-hydroxyvitamin D concentration of 20–29 ng/mL). Only, 4 women (2.2 %) were sufficient (25-hydroxyvitamin D concentration of ≥ 30 ng/mL).

Age, depression score, amount of sun exposure and seasons were compared between women with 25-hydroxyvitamin D concentrations of <20 ng/mL and who had concentrations of ≥ 20 ng/mL (Table 2).

**Table 2:** Means age and depression score, sun exposure and seasons in the participants with 25-hydroxyvitamin D concentrations of <20 and ≥ 20 ng/mL

Variables	Serum 25 (OH) D (ng/ml)		*p*-value
	<20	≥20	
Age (years), mean ± sd	28.45 ± 4.62	25.32 ± 4.39	0.31
Depression score at baseline, mean ± sd	8.34 ± 3.82	9.37 ± 3.94	0.31
Sun exposure, N (%)			<0.001
< 60 m	105 (89.7 %)	8 (44.4 %)	
≥ 60 m	12 (10.3 %)	10 (55.6 %)	
Season, N (%)			0.05
Spring	24 (75 %)	8 (25 %)	
Summer	16 (88.9 %)	2 (11.1 %)	
Winter	94 (91.3 %)	9 (8.7 %)	

At childbirth, 25-hydroxyvitamin D had a mean value of 14.64 ± 8.60, with a minimum and maximum value 4 and 51.10 ng/mL, respectively. Ninety-nine women (76.2 %), 25 women (19.2 %), and six women (4.6 %) had 25-hydroxyvitamin D concentrations of < 20, 20–29 and ≥ 30 ng/mL, respectively. The between groups comparisons of 25-hydroxyvitamin D concentrations were presented in Table 3.

**Table 3:** Between groups comparisons of depression scores and 25(OH)D concentrations

Variables	mean ± sd			Depression score, N (%)						
	Vitamin D group	Control group	*P*-value	Vitamin D group			Control group			
				<9	9–13	>13	<9	9–13	>13	*P*-value*
Depression score at baseline	8.44 ± 3.89	8.64 ± 3.81	0.747	37 (49.3)	38 (50.7)	0 (0)	33 (42.3)	45 (57.7)	0 (0)	0.383
Depression score at 38–40w gestation	6.17 ± 3.47	7.77 ± 3.92	0.01	49 (72.1)	18 (26.5)	1 (1.5)	37 (50.7)	30 (41.1)	6 (8.2)	0.024
Depression score at post partum (4w)	4.59 ± 3.29	7.36 ± 4.27	<0.001	58 (90.6)	6 (9.4)	0 (0)	43 (59.7)	23 (31.9)	6 (8.3)	<0.001
Depression score at postpartum (8w)	4.19 ± 3.76	7.18 ± 3.99	<0.001	57 (89.1)	7 (10.9)	0 (0)	46 (63.9)	22 (30.6)	4 (5.6)	0.001
				Categorized 25(OH)D, N(%)						
				<20	20–29	≥30	<20	20–29	≥30	*P*-value*
25(OH)D at baseline (ng/mL)	12.84 ± 7.91	11.89 ± 6.40	0.63^a^	65 (86.7)	7 (9.3)	3 (4)	69 (88.5)	8 (10.3)	1 (1.3)	0.61
25(OH)D at delivery (ng/mL)	17.46 ± 10.09	12.07 ± 5.98	0.001^a^	41 (66.1)	15 (24.2)	6 (9.7)	58 (85.3)	10 (14.7)	0 (0)	0.006

^*^Fisher’s Exact Test

^a^Mann-Whitney *U* test

The mean depression score of 184 participants was 9.20 ± 4.44 at baseline. Additionally, a minimum and maximum value of 0 and 24, median 9.50, and mode 13 were observed. Out of which, 79 women (42.9 %) scored <9, 90 women (57.1 %) scored 9–13 and 15 women (8.2 %) scored > 13. The low risk women for depression (a depression score of <9) were compared to the high risk women (a depression score of > 13) concerning age (26.91 ± 4.36 vs. 25.80 ± 4.74) and 25-hydroxyvitamin D concentration (11.26 ± 6 vs. 13.25 ± 7.96). They did not differ concerning the two above-mentioned variables, *P* = 0.09 and *p* = 0.13, respectively. The mean depression scores in the nulliparous and multiparous women were 8.58 ± 3.53 and 8.48 ± 4.29, respectively with no significant difference (*p* = 0.86). In our study, measures of reliability, including a Cronbach’s alpha and a test- retest correlation were 0.86 and 0.92, respectively.

After eliminating some of the mothers who scored > 13, 153 women continued their participation in the study. Their mean depression score changed to 8.54 ± 3.84. The median was nine and the mode was 13. Among them, 70 women (45.8 %) scored <9 and 83 women (54.2 %) scored 9–13. At baseline, using Pearson’s correlation coefficient, no relations were observed between the 25-hydroxyvitamin D concentrations and depression scores (*r* = 0.13, *p* = 0.09). There was no significant difference between the two study groups in relation to the baseline depression scores (0.74). While, the mean depression scores were significantly lower in the vitamin D group than the control one at 38–40 weeks of gestation (*p* = 0.01) also, at 4 and 8 weeks after birth (*p* > 0.001) (Table 3). Depression scores were divided into three categories, <9, 9–13, and >13, and using the Fisher’s exact test, the comparisons between the two groups were conducted. Results showed that in the vitamin D group the number of participants with depression scores of <9 were more than the control group at 38–40 weeks of gestation also, at 4 and 8 weeks after birth (Table 3).

**Fig. 2:**
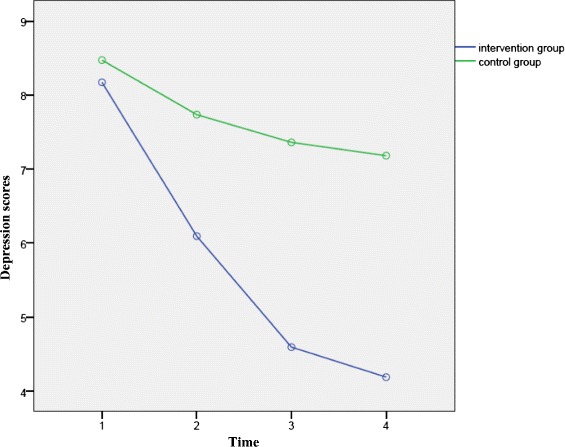
Within groups comparison related to depression scores: The results showed, between the two groups, the difference regards to over-time reduction of depression scores was significant (*p* < 0.001)

The repeated measures analysis showed that the control group had gone through significant changes with regards to depression scores (*p* < 0.001) (Fig. 2). In pairwise comparison, the baseline depression score and the scores at 38–40 weeks were not different significantly (*p* = 0.142). However, the scores at 4 and 8 weeks after birth were lower than the baseline depression scores ((*p* = 0.048 and *p* = 0.014, respectively).

The vitamin D group also had gone through changes with regards to their depression scores (*p* < 0.001) (Fig. 2)*.* Pairwise comparisons showed that the scores at 38–40 weeks, 4 and 8 weeks postpartum were significantly lower than the baseline depression scores (*p* < 0.001).

However, the depression scores at 4 and 8 weeks after birth were not different significantly (*p* = 0.217).

The participants were divided in to two subgroups, the nulliparous and multiparous subgroups and data was analyzed once more. The results showed that in both subgroups, the vitamin D group had statistically significant higher 25-hydroxyvitamin D concentrations at childbirth and lower depression score at 4 and 8 weeks postpartum than the control group (Table 4). While, any statistically significant differences were not observed regarding to depression scores at 38–40 weeks of gestation (Table 4).

**Table 4:** 25(OH)D concentrations and depression scores in nulliparous and multiparous subgroups

Variables Mean (sd)	Nulliparous				Multiparous			
	Vitamin D group	Control group	N	*p*-value	Vitamin D group	Control group	N	*p*-value^*^
25(OH)D at baseline (ng/mL)	13.13(6.91)	13.25 (6.55)	41/51	0.93	12.49 (9.08)	9.31 (5.34)	34/27	0.15
25(OH)D at delivery (ng/mL)	18.40 (11.38)	12.49 (6.29)	33/43	0.01	16.58 (8.54)	11.36 (5.47)	28/25	0.02
Depression score at baseline	8.41(3.52)	8.73 (3.57)	41/51	0.67	8.47 (4.35)	8.48 (4.30)	34/27	0.99
Depression score at 38-40w gestation	6.47(3.43)	7.98 (3.74)	38/46	0.06	5.80 (3.62)	7.44 (4.28)	30/27	0.12
Depression score at postpartum (4w)	4.59(3.24)	7.27 (4.04)	34/45	0.002	4.60 (3.40)	7.52 (4.70)	30/27	0.01
Depression score at postpartum (8w)	4.18(3.79)	7.24 (3.79)	34/45	0.001	4.20 (3.79)	7.07 (4.37)	30/27	0.01

^*^Mann–Whitney *U* test

We examined the correlation between change in vitamin D concentrations and change in depression scores at 38–40 weeks of gestation, 4 and 8 weeks postpartum. We did not observed any correlation at 38–40 weeks (*r* = 0.14, *p* = 0.29) or at 4 and 8 weeks postpartum (*r* = 0.1, *p* = 0.43) (*r* = −0.02, *p* = 0.83) respectively.

## Discussion

This clinical trial evaluated the effect of 2000 IU of vitamin D_3_ supplement per day during late pregnancy on perinatal depression. According to our knowledge, this study was the first trial held on vitamin D and perinatal depression. Results showed that at baseline, in 72.8 % of the participants, serum 25-hydroxyvitamin D concentrations were lower than20 ng/mL. They were suffering from vitamin D deficiency based on new perspective towards normal serum vitamin D concentration [46]. Although 67.3 % of the blood samples were taken in the winter, 81 % of the participants were exposed to sunlight or had outdoor activities for at least 15 min per day. Considering the factors affecting vitamin D production such as clothing type, skin color and the time of day being exposed to the sun [48–50], these results are justifiable.

Vitamin D deficiency is a widespread global problem, which even can observe in the countries which get enough sunlight [49, 51]. The high prevalence of vitamin D deficiency in Iran was showed in numerous studies. In a study by Hovsepian et al., in men and women with average age of 41.4 years in Isfahan, 45 % of men and 52.4 % of women had vitamin D concentrations lower than 20 ng/mL ([52]). Saki et al., showed high prevalence of vitamin D deficiency in southern Iranian children, more than 80 % were deficient [53]. Also, Omani et al. found vitamin D deficiency in 52.9 % of women aged 20–74 years in Shiraz, Iran [54]. In a study by Hashempoor et al., in Tehran, 67.1 % of the men and women had vitamin D concentrations lower than 25 ng/mL [55]. In a study by Kazemi et al., in 67 mothers with term birth in Zanjan, 86 % in the winter and 46 % in the summer had vitamin D levels lower than 25 ng/. The total mean was 19.4 ± 3.9 ng/mL [56]. In the present study, the mean vitamin D concentrations in vitamin D group were significantly higher after taking 2000 IU of vitamin D supplementation per day for at least 8 weeks. Nonetheless, only 9.7 % in the vitamin D group achieved serum concentrations higher than 30 ng/mL. In contrast, in the previous studies 2000 IU vitamin D supplementation was more sufficient to increase serum 25-hydroxyvitamin D concentrations up to the adequate amount (≥32 ng/mL) [18, 57, 58]. In those studies, vitamin D supplementations started at the end of the first trimester or at the beginning of the second trimester and their participants had higher initial 25-hydroxyvitamin D concentrations compared to the participants in our study. Ruth et al. in their study used a dosage of 14,000 IU vitamin D supplement per week, equal to 2000 IU per day, started at 27–30 weeks of gestation. Of total, 89 % achieved vitamin D concentration of higher than 20 ng/mL and 56 % achieved vitamin D concentration of higher than 32 ng/mL [57]. In Tehran, Taheri et al. gave 2000 IU of vitamin D per day to non-pregnant women in form of oral drops for 105 days. Initial mean 25-hydroxyvitamin D level was 9.35 ± 6.33 ng/mL in the control group and was 10.05 ± 7.38 ng/mL in the intervention group. After intervention, the two groups were different significantly. Almost half of the intervention group achieved concentrations of 30 ng/mL [59]. Maternal 25-hydroxyvitamin D transfers to the fetus by the placenta. Therefore, it is justifiable that in our study, vitamin D group had lower 25-hydroxyvitamin D concentration compared to those were in the study by Taheri et al.

Considering cut-off point of ≥9, data analysis showed that a lot of participants (more than 50 %) were susceptible to prenatal depression at 26–28 weeks. When using cut-off point of >13, susceptibility to prenatal depression decreases to 8.2 %. In previous studies, the prevalence of antenatal depression had been studied, using various tools and cut-off points. In a cohort study by Evans et al. 11.8 % at 18 weeks and 13.5 % at 32 weeks were depressed with the threshold score of 13 or higher [60]. In a study in a private center in Brazil, the prevalence of antenatal depression was 19.6 % with the Beck tool [61]. In a study by Shackle et al. in Oslo, prevalence of depression at 28 weeks was 13 % in all of samples. Depression was reported in 8.6 % of European mothers, in 19.5 % of Middle Eastern mothers, and in 17.5 % of South Asian mothers. The threshold of ≥10 was used based on the EPDS [62]. However, the EPDS has been widely used in screening, not providing a diagnosis, for perinatal depression, it has been reported that its score is highly correlated with physician diagnostic opinion. Several studies have been shown that, with cutoff score of 12–13, this scale develops into more indicative in identifying real depression in women [36, 37, 42]. The participants who had depression scores of >13 were exited from this clinical trial and referred to psychiatrist for further assessment and treatment, because, the nature of our study was preventative than management.

In our study, antenatal depression score had no association with the baseline 25-hydroxyvitamin D concentration. While in a study by Cassidy- Bushrow et al., in African-American women, an inverse relationship was observed between maternal 25-hydroxyvitamin D concentration at the early stages of pregnancy and antenatal depression. There was an inverse relationship between the vitamin D logarithm and depression scores, as by adding one unit to the vitamin D concentration logarithm, equal to 2.72 ng/mL, a 46 % reduction in the depression scores occurred [63]. In another study in US, in 498 pregnant mothers with the mean gestational age of 15 weeks, generally there was an inverse relationship between 25-hydroxyvitamin D concentration and depression level. With 1 ng/mL reduction in 25-hydroxyvitamin D concentration, the anxiety and depression levels increased 0.043 and 0.040, respectively [64]. It is worth mentioning that their tools for determining depression were different than ours. In our study, nearly 70 % of the mothers had vitamin D deficiency. It was a possible reason that we did not observe any relationship between antenatal depression score and 25-hydroxyvitamin D concentration. Also in our study, selection of participants at the beginning was through convenience method. Surprisingly, the change in vitamin D concentration (baseline to childbirth) was not correlated with changes in depression scores from baseline to 38–40 weeks of gestation or to 4 and 8 weeks postpartum.

The present trial showed that consuming 2000 IU vitamin D_3_ per day in late pregnancy was effective in decreasing depression scores in perinatal period. The vitamin D and control groups were similar regarding to general characteristics except for two variables: unplanned pregnancy and using other supplements out of the study’s protocol. Unplanned pregnancy was more frequent in the vitamin D group than the control group. Previous studies reported that unplanned pregnancy is a risk factor for perinatal depression. Nonetheless, in our study, the vitamin D group had significantly lower depression scores after intervention. The effectiveness of calcium supplementation on prevention of perinatal depression has been shown in a previous study [65]. The control group consumed extra supplements such as calcium more frequently, nonetheless this group had significantly higher depression scores compared to vitamin D group.

Previous clinical trials, in which vitamin D supplementations were consumed during pregnancy [18, 58], have not evaluated the antenatal and postnatal depression disorders. However in a cross-sectional study in Japan, higher dietary vitamin D intake was independently associated with a lower prevalence of depressive symptoms during pregnancy [66].

On the other hand, the effect of vitamin D on depression in non-pregnant people are not entirely clear. Some clinical trials in non-pregnant people showed that vitamin D was effective in decreasing depression level. In a study by Mozafari- Khosravi et al., 300,000 and 150,000 IU vitamin D were administered by injection to the two groups who suffered depression and the third group did not receive vitamin D. High doses of vitamin D were effective in decreasing depression levels after 3 months [30]. In another study, 20,000 and 40,000 IU vitamin D per week were given to the obese and overweight people for one year. Comparing to the control group, depression level decreased in both intervention groups [67]. In contrast, in another study 40,000 IU vitamin D per week was used for 6 months, but vitamin D was not effective in decreasing depression level compared to the control group [68]. Dean et al. found that 5000 IU vitamin D per day for 6 weeks had no effect on men and women’s cognitive and emotional function [69].

Vitamin D can affect brain function in several ways. The existence of receptors for the active form of vitamin D (1, 25(OH)2D) in the brain induces a direct effect. The essential enzymes for vitamin D hydroxylation are found in the brain that can produce calcitriol. Its indirect effects are attributed to vitamin D’s effects on improvement of muscle activities. As a result of this improvement, physical activity increases and this have positive effects on health. An increase in vitamin D can cause parathyroid hormones to decrease. An increase in this hormone can lead to brain dysfunctions [70, 71]. On the other hand, cytokines are messengers produced by immune cells which affect all tissues, including brain. Nowadays, we know that brain cells such as microglia and astrocytes, can secrete cytokines. Besides, activated lymphocytes can cross the blood–brain barrier and secrete cytokines in the brain [72]. Vitamin D may act as an anti-inflammatory agent in the brain by keeping harmful pro-inflammatory cytokines in check. Vitamin D deficiency has been linked to pro-inflammatory disease. It is also possible that as a result of vascular changes in pregnancy the maternal cerebral environment is more vulnerable to inflammation perhaps, explaining in part the link between vitamin D and depression in pregnancy. In light of the inflammatory nature of depression and the strong role of vitamin D as an anti-inflammatory and immunomodulator, inflammation may mediate the relationship between vitamin D deficiency and depression during pregnancy [73, 74].

Previous studies have suggested that perinatal depression is more frequent and serious in primiparous women than multiparous women [75, 76]. It was interesting to investigate trend of vitamin D effectiveness in the two primiparous and multiparous subgroups. The findings were similar to the overall population except one thing. As, in the two subgroups, the depression scores were not significantly lower in vitamin D group compared to control group at 38–40 weeks. Staying on vitamin D consumption until childbirth resulted in higher serum 25-hydroxyvitamin D concentration that induced the effect on postnatal depression exceeding than prenatal depression. A difference in nature of antenatal depression compared to postnatal depression can reflect another possible reason for achieving this result.

Depression scores decreased in the both our groups over time which indicates that depression could be more critical during pregnancy than postpartum period.

The pleasant feeling of motherhood can probably play a role in decreasing depression specially in primiparous mothers [77]. During pregnancy, women are worried about risks to their own and the fetus health. Fear of labor pain is another concern of the modern-day mothers [78]. Therefore, a decreased perinatal depression level is justifiable. On the other hand, the difference between the two groups was meaningful concerning over-time reduction of depression. Meaning that over time, depression scores decreased much more in the vitamin D group in comparison to control group and this shows the possible effects of the vitamin D_3_ supplementation. Another finding of this study was that vitamin D_3_ supplementation was effective in decreasing depression level in both nulliparous and multiparous subgroups.

### Limitations

Vitamin D consumption by mothers was merely controlled through reminders during prenatal care visits or over the phone. Therefore, the participants’ honesty was one of this research limits. The participants were selected from one prenatal clinic. This group of participants may not be representative of the target population. Since mothers with depression level of >13 were excluded from this study, the results can not extend to mothers with high levels of depression. Also, since more than 95 % of the mothers had lower than 30 ng/mL serum 25-hydroxyvitamin D concentration, it is not clear if the same results would be observed in mothers with higher levels of 25-hydroxyvitamin D. Therefore, we suggest further clinical trials, using 2000 IU vitamin D on a larger population, mothers with high levels of depression, and high serum 25-hydroxyvitamin D concentrations. The last weakness of this study was that it had the statistical authority to identify differences in depressive symptoms, but not in the diagnoses of major depressive disorder.

## Conclusion

In the present study, it was observed that 2000 IU vitamin D_3_ per day for at least 8 weeks during late pregnancy, can be effective in decreasing perinatal depression levels (at 38–40 weeks of gestation also, at 4 and 8 weeks after birth). Most pregnant mothers tolerated the 2000 IU well without any adverse effects and just a few of them temporarily complained about nausea.

## Additional file

Additional file 1: Dataset supporting the conclusions of this article. (XLSX 31 kb)
